# Banning Psychoactive Substances: A Slippery Slope

**DOI:** 10.1016/j.ebiom.2015.07.016

**Published:** 2015-07-16

On May 28, 2015, the Psychoactive Substances Bill was introduced in the UK House of Lords. Ostensibly meant to curtail so-called “legal highs” — newly-synthesized chemical intoxicants that are not covered by current drug laws — the bill would impose a blanket ban on “any substance that […] affects the person's mental functioning or emotional state”. While the bill's objective of preventing personal injury and public harm is noble, it and similar laws in other nations could have unintended consequences, including hindrance of an already ailing psychiatric research and drug development pipeline.

The prominence of new psychoactive substances (NPSs) has risen over the past two decades, on the backs of clever chemists, marketers, and internet entrepreneurs. Many NPSs are derivatives or analogs of other, often illegal, psychoactive compounds. To circumvent the United Nations' 1971 Convention on Psychotropic Substances, which influenced many domestic policies, NPSs are sold at shops as not for human consumption under the guise of plant food, bath salts, and incense, complete with appealing packaging and names. A prolific internet drug trade has also contributed to the emergence of roughly one NPS per week since the late 2000s. Keeping pace with the NPS industry's agility imposes serious burden on the legal system.

Regulation of NPSs is necessary. Daily, they harm users who are unaware of, or cannot predict, how an untested drug will affect their bodies. Mimicking narcotics like cocaine, opiates, amphetamines, and cannabis, NPSs have been known to cause hypertension, heart palpitations, seizures, blindness, Parkinsonism, seizures, and death. Laws like the Psychoactive Substances Bill aim to disrupt manufacturing and distribution of NPSs, preventing them from falling into the public's hands. In the real world, though, drug law repercussions on public interests are hard to predict.

These laws would also impact the research community. For example, under the US Drug Enforcement Agency's scheduling system, any drug that has no currently accepted medical use — such as LSD (acid), MDMA (ecstasy), and psilocybin (mushrooms) — falls under Schedule I. Obtaining a license to perform research using these drugs requires considerable money and time, not to mention institution-level ethics review, dissuading many scientists from the bother of research on psychoactive substances. In one example, the burgeoning field of LSD-assisted psychotherapy all but ended in the 1970s when new laws made the drug nearly impossible for researchers to obtain.

The contradictions among laws and government-funded research policies have left many scientists confused. On the one hand, current and proposed drug laws that apply to the public also apply to researchers. On the other hand, multi-billion dollar projects, like Europe's Human Brain Project, the US's BRAIN Initiative, and the World Health Organization (WHO)'s Mental Health Action Plan 2013–2020, incentivize neuroscience research aimed at tackling the global health epidemic of mental illness. Affecting over 400 million people worldwide, mental illness is the second-leading cause of otherwise healthy days lost to disability. With ever-increasing incidence rates of major depressive disorder, anxiety disorders, schizophrenia, autism spectrum disorders, and dementia, neuropsychiatric disorders are poised to become the most economically burdensome diseases on the planet within a decade.

Existing research programs are lagging behind the growing need, with many pharmaceutical companies divesting from neuropsychiatric research, leaving a drought in the drug development pipeline. Despite current obstacles in psychiatric research, though, promising results are arising from early preclinical testing of psychoactive substances. MDMA has been shown effective in the treatment of post-traumatic stress disorder and social anxiety. LSD and psilocybin can assist in alcohol addiction rehabilitation. Cannabis, illegal in many countries, has been used to treat an array of disorders, including multiple sclerosis, chronic pain, nausea, and epilepsy. Notably, legalization of marijuana for medical use in some US states has not affected overall rates of cannabis use among adolescents. Expanding drug laws, without specific concessions to scientists, will only reinforce existing barriers to research and prevent potentially life-changing therapies from being realized.

In July 2013, instead of a blanket ban on NPSs, New Zealand enacted a different approach: testing. Any new molecule that is chemically related to or intended to have similar physiological effects as another psychoactive compound is subject to the same rigorous testing as other pharmaceuticals before being publically available, with manufacturers and experimenters being strictly regulated. Under these policies, NPS availability has dropped 75%. To date, no NPS has been licensed in New Zealand, but the pragmatic, scientific approach of the law seems admirable. Can we outlaw or restrict access to a drug for having no medical use and for affecting the user's mental functioning or emotional state without explicitly researching those concerns?

The Psychoactive Substances Bill inadvertently exposes numerous paradoxes and contradictions in the way governments, society, and individuals think about drugs. Policymakers would need to re-evaluate why amphetamines can be used to treat Attention Deficit-Hyperactivity Disorder while methamphetamine is illegal. Or why the cathinone buproprion can be prescribed for major depressive disorder but its analog, mephedrone, is banned. And why tryptamines are available for treatment of migraines while the related LSD and psilocybin are Schedule I drugs. Explicit clarity in legislation would be required in exempting current psychoactive substances, including some opioid analgesics with both high therapeutic value and abuse potential, along with alcohol and tobacco, which kill 2.5 and 6 million people per year, respectively.

A second reading of the Psychoactive Substances Bill began on June 30, 2015. Skeptics warned “of a lack of research underpinning policy development” that had policymakers operating “effectively blind.” While new legislation to address developing concerns on psychoactive substances is mandatory, so must be the careful measurement of their harms and potential benefits. The overwhelming burden of mental illness demands and deserves a thoughtful, non-reactionary approach in managing the molecules that could be tomorrow's psychiatric medicines.

*EBioMedicine*

